# Lectin-Dependent Enhancement of Ebola Virus Infection via Soluble and Transmembrane C-type Lectin Receptors

**DOI:** 10.1371/journal.pone.0060838

**Published:** 2013-04-02

**Authors:** Matthew Brudner, Marshall Karpel, Calli Lear, Li Chen, L. Michael Yantosca, Corinne Scully, Ashish Sarraju, Anna Sokolovska, M. Reza Zariffard, Damon P. Eisen, Bruce A. Mungall, Darrell N. Kotton, Amel Omari, I-Chueh Huang, Michael Farzan, Kazue Takahashi, Lynda Stuart, Gregory L. Stahl, Alan B. Ezekowitz, Gregory T. Spear, Gene G. Olinger, Emmett V. Schmidt, Ian C. Michelow

**Affiliations:** 1 Programs of Developmental Immunology, Department of Pediatrics, Massachusetts General Hospital, Boston, Massachusetts, United States of America; 2 Virology Division, United States Army Medical Research Institute of Infectious Diseases, Fort Detrick, Frederick, Maryland, United States of America; 3 Department of Immunology and Microbiology, Rush University Medical Center, Chicago, Illinois, United States of America; 4 Victorian Infectious Diseases Service, Royal Melbourne Hospital, Parkville, Victoria, Australia; 5 Australian Animal Health Laboratory, Commonwealth Scientific and Industrial Research Organisation (CSIRO) Livestock Industries, Geelong, Victoria, Australia; 6 Department of Medicine, Boston University School of Medicine, Boston, Massachusetts, United States of America; 7 New England Primate Research Center, Southborough, Massachusetts, United States of America; 8 CETRI, Brigham and Women's Hospital, Boston, Massachusetts, United States of America; 9 Harvard Medical School, Boston, Massachusetts, United States of America; Metabiota, United States of America

## Abstract

Mannose-binding lectin (MBL) is a key soluble effector of the innate immune system that recognizes pathogen-specific surface glycans. Surprisingly, low-producing MBL genetic variants that may predispose children and immunocompromised individuals to infectious diseases are more common than would be expected in human populations. Since certain immune defense molecules, such as immunoglobulins, can be exploited by invasive pathogens, we hypothesized that MBL might also enhance infections in some circumstances. Consequently, the low and intermediate MBL levels commonly found in human populations might be the result of balancing selection. Using model infection systems with pseudotyped and authentic glycosylated viruses, we demonstrated that MBL indeed enhances infection of Ebola, Hendra, Nipah and West Nile viruses in low complement conditions. Mechanistic studies with Ebola virus (EBOV) glycoprotein pseudotyped lentiviruses confirmed that MBL binds to *N*-linked glycan epitopes on viral surfaces in a specific manner via the MBL carbohydrate recognition domain, which is necessary for enhanced infection. MBL mediates lipid-raft-dependent macropinocytosis of EBOV via a pathway that appears to require less actin or early endosomal processing compared with the filovirus canonical endocytic pathway. Using a validated RNA interference screen, we identified C1QBP (gC1qR) as a candidate surface receptor that mediates MBL-dependent enhancement of EBOV infection. We also identified dectin-2 (CLEC6A) as a potentially novel candidate attachment factor for EBOV. Our findings support the concept of an innate immune haplotype that represents critical interactions between MBL and complement component C4 genes and that may modify susceptibility or resistance to certain glycosylated pathogens. Therefore, higher levels of native or exogenous MBL could be deleterious in the setting of relative hypocomplementemia which can occur genetically or because of immunodepletion during active infections. Our findings confirm our hypothesis that the pressure of infectious diseases may have contributed in part to evolutionary selection of MBL mutant haplotypes.

## Introduction

Mammalian viruses have co-evolved with the host's immune machinery because viruses are obligate intracellular parasites. The outcome of host-virus interactions rests on a delicate balance between the host's defenses and the damage caused by viruses [1]. In addition, viruses have developed counter-adaptation strategies to escape immune defenses by evading, suppressing or manipulating the host's immune response. Consequently, favorable genetic traits in both hosts and viruses have been naturally selected over time [2].

Human mannose-binding lectin (MBL), a prototypic soluble calcium-dependent (C-type) lectin, is a key first line host defense molecule and immune regulator. MBL collaborates with a network of host defenses such as complement components and diverse cellular receptors such as C-type lectin, scavenger, and complement receptors, integrins, and Toll-like receptors (TLR) to prime long-lived adaptive immunity [3], [4]. MBL recognizes specific molecular patterns of oligosaccharides including D-mannose, L-fucose, glucose, and *N*-acetylglucosamine that decorate the surface of many viruses such as Ebola virus (EBOV), Hendra and Nipah viruses, HIV-1, HIV-2, influenza viruses, and severe acute respiratory syndrome coronavirus [5], [6]. MBL oligomers form complexes with MBL-associated serine protease (MASP) dimers which activate the lectin complement pathway and enhance opsonophagocytosis of pathogens [5]. The average human MBL serum concentration is 1.5 µg/ml (range, 0 to >5 µg/ml) [7] but up to 30% of the human population has levels <500 ng/ml, which may be associated with increased susceptibility to infections in young children and immunocompromised individuals [5]. Interindividual variability of MBL concentrations is caused by structural variant alleles (*B*, *C*, and *D*) in exon 1 of the *MBL2* gene which disrupt assembly of MBL high-order oligomers and lead to reduced MBL concentrations and activity. Single nucleotide polymorphisms in the promoter region (*H*/*L*, *X*/*Y*), 5′ untranslated region (*P*/*Q*) and other regulatory sites also modify serum concentrations. Together, these sequence variations define at least 7 common human MBL haplotypes that dictate MBL concentrations and complement-activating capacity. MBL variant alleles are present in approximately 20% to 50% of individuals. Higher rates occur in indigenous people from Africa and South America [8]. The reason for such high rates of sequence variations is an unresolved controversy [9], [10]. It has been postulated that a high-producing ancestral haplotype (*A* allele) evolved into multiple low MBL-producing haplotypes because of heterotic balanced selection in which individuals carrying structural and regulatory heterozygous polymorphisms had a survival advantage [11]. It is not known whether viral infections exerted such selective pressure over time.

EBOV is a non-segmented, negative-strand RNA virus of the order *Mononegavirales* and family *Filoviridae* that can cause rapidly fatal viral hemorrhagic fevers in part by dysregulating the innate immune system. Its highly glycosylated viral envelope glycoprotein (GP_1,2_) mediates receptor binding (GP_1_) and virus-host cell membrane fusion (GP_2_) by targeting ubiquitous lectins and other molecules expressed by macrophages, dendritic cells and endothelial cells [12], [13]. We previously demonstrated that recombinant human MBL (rhMBL) binds EBOV GP_1,2_ (referred to as GP) lentiviral virion-like particles and wild-type-like EBOV in a specific and dose-dependent manner [6], [14]. Treatment of mice infected with 3,000×LD_50_ native EBOV using supraphysiological dosages of rhMBL had a protective effect which required intact complement component 3 (C3) function [15]. Taken together, these data suggested that MBL and the lectin complement pathway can influence the course of EBOV infections.

Viruses infect cells by co-opting existing cellular structures or functions that are responsible for endocytosis of fluid and small particles, cell-cell recognition, ion transport, and binding to the extracellular matrix. First, viruses bind to attachment factors and/or cognate receptors which help concentrate the virus on the cell surface. Upon binding, receptors then promote endocytosis or trigger direct fusion of viral and host cell-membranes [16]. EBOV is internalized primarily by macropinocytosis [17] whereas certain other viruses enter cells via clathrin-coated pits, caveolar/lipid-raft structures, or clathrin− and caveolin/raft-independent mechanisms [16]. In general, the specificity of receptor binding determines cell tropism, and the type of receptor engaged by the virus determines the choice of endocytic pathway. Notably, EBOV has very broad cell tropism, especially later in the course of infection, and it may bind to multiple attachment factors, notable among which are numerous lectins (DC-SIGN/L-SIGN, MGL [CLEC10A], LSECtin [CLEC4G]) and Niemann-Pick C1 endosomal membrane protein. In addition, Tyro3 family members (Axl, Dtk, Mer) and β1 integrins which are widely expressed have been implicated in Ebola-GP mediated cell entry [13], [18]. T-cell immunoglobulin and mucin domain 1 (TIM-1) was recently proposed as an epithelial receptor that binds the EBOV GP_1_ receptor binding region [19] but the cognate receptor(s) of monocytes, macrophages and dendritic cells have yet to be identified.

A variety of viruses including EBOV, Dengue viruses, West Nile virus (WNV), HIV-1, Coxsackie B virus and Ross River virus exploit antibodies or activated complement components to enhance entry into target cells [20], [21], [22]. This process, called antibody-dependent enhancement (ADE), starts with cross-linking of virus-antibody or virus-antibody-complement complexes to Fcγ (CD32) or complement receptors, respectively [20], [21], [23]. The virus may then be coupled to its cognate receptor on the cell surface, thereby facilitating entry. It is noteworthy that viral specific antibodies can either neutralize or enhance productive EBOV and WNV infection depending on the antibody concentration and class, complement component concentration, type of cells, density of epitopes, and effector mechanisms mediated by the crystallizable fragment (Fc) of the antibody [22], [24], [25]. It has also been postulated that the rapid spread of EBOV to secondary target cells (e.g. hepatocytes and endothelial cells) occurs by means of ADE via widely distributed C1q receptors [22].

MBL has been described as an “ante-antibody” which represents a primitive non-clonal form of humoral immunity and which evolutionarily predated antibodies [26]. Analogous to antibodies, MBL mediates opsonophagocytosis and it can bind to cellular receptors via its collagenous tail [27]. In addition, MBL has structural and functional homology with C1q which binds cognate cellular receptors and/or activates the complement pathway via actions of serine proteases [28]. Given the functional similarities between MBL and either antibodies or C1q, we considered that a novel MBL receptor-mediated mechanism for enhancing infection by EBOV and other viruses whose virions are highly glycosylated might explain some of the heterotic balanced selection that may contribute to the evolutionary divergence of human MBL haplotypes.

Given our proposed model that lectins might enhance EBOV infection, we investigated the possibility that human MBL could enhance infection of human cells by EBOV GP-pseudotyped lentiviruses, wild-type-like EBOV and other glycosylated virions in low complement conditions. After initial confirmation of our model, we pursued mechanistic studies of the dynamics of MBL-mediated endocytosis and identified candidate MBL and viral receptors using an RNA interference (RNAi) approach.

## Materials and Methods

### Ethics statement

We obtained written informed consent from eligible healthy adult volunteers for blood samples according to a protocol approved by the Massachusetts General Hospital Institutional Review Board.

### Reagents and antibodies

All reagents were purchased from Sigma-Aldrich (St. Louis, MO) except as listed. Jasplakinolide was purchased from EMD Biosciences (San Diego, CA) and Latrunculin B from Enzo Life Sciences International (Plymouth Meeting, PA). Wild-type-like cyanovirin-N (P51G mutant) was kindly donated by E. Matei [29]. RhMBL was provided by Enzon Pharmaceuticals (Bridgewater, NJ)[30]. Notably, rhMBL is similar in structure and function to native human MBL but forms predominantly high molecular mass multimers >350 kDa [30]. Complement component C2-depleted serum was purchased from Quidel (San Diego, CA). Recombinant human C2 and interleukin-4 (IL-4) were purchased from R&D Systems (Minneapolis, MN). N-glycosidase F (PNGase F) and endoglycosidase H were from New England Biolabs (Ipswich, MA). Anti-hMBL antibody HYB 131-01 (BioPorto, Gentofte, Denmark) was used for mannan-binding and complement component C4 cleavage assays. The anti-human MBL neutralizing monoclonal antibody, 3F8, was produced by G.L. Stahl [31]. The isotypic control IgG1 was obtained from Beckman Coulter (Hebron, KY). Puromycin and alamarBlue cell viability reagent (resazurin) were from Invitrogen (Carlsbad, CA). Thermolysin (Sigma-Aldrich) was derived from *Thermoproteolyticus rokko*. Biotinylated *Hippeastrum* hybrid (amaryllis) lectin was obtained from Vector Laboratories (Burlingame, CA).

### Cells and culture

Cell lines were purchased from the American Type Culture Collection (ATCC, Manassas, VA) and maintained in a humidified 37°C incubator with 5% CO_2_ in the following media: HEK293F and Vero E6 cells, Eagle's Minimum Essential Medium (EMEM); HEK293T cells, Dulbecco's Modified Eagle Medium (DMEM) with 1% Hepes; HEK 293S *N*-acetylglucosaminyltransferase I^−^ (GnTI^−^) cells, DMEM:F12, and THP-1 cells and peripheral blood mononuclear cells (PBMC), RPMI-1640. Media were supplemented with 10% fetal bovine serum (Invitrogen), 100 U/ml penicillin, and 100 µg/ml streptomycin. Of note, HEK293F cells adhere to tissue culture microplates (Corning) when incubated with FBS-supplemented media. PBMC were isolated from fresh single-donor buffy coat samples from the Massachusetts General Hospital (MGH) Blood Bank using Vacutainer CPT Cell Preparation Tubes with sodium citrate (BD, Franklin Lakes, NJ) according to the manufacturer's instructions. 6×10^7^ cells were seeded in RPMI-1640 with 10% FBS in a 10 cm tissue-culture dish at 37°C for 3 hours after which non-adherent cells were removed by washing twice with RPMI-1640. Adherent PBMC were detached with a cell scraper and seeded at 2.5×10^5^/well in a 96-well tissue-culture plate in RPMI-1640 with 10% FBS and 50 ng/ml M-CSF to induce differentiation of monocyte-derived macrophages (day 0). Then cells were incubated at 37°C, fresh media (50 µl) and M-CSF (50 ng/ml) were added on day 4, cells were infected on day 7, and assayed for infection on day 10.

### MBL functional assays and MBL2 genotyping

Mannan-binding and C4 cleavage assays were performed as described elsewhere [6]. We isolated genomic DNA from 3 ml whole blood obtained from 35 ethnically diverse, consenting healthy volunteers according to the manufacturer's instructions (Gentra Puregene Blood Kit, Qiagen, Valencia, CA). Primers and PCR reaction conditions for *MBL2* genotyping were adapted from Steffensen et al. [32]. Modifications to the protocol are described in Methods S1.

### Virus preparation

#### Pseudotyped viruses

HIV-EBOV GP, HIV-EBOV-ΔGP NTDL6 (217 amino acids were deleted from the mucin-rich region downstream of the EBOV binding region), HIV-vesicular stomatitis virus (VSV)-G, and HIV-envelope (*env*) negative (gp120^−^) virions, which encoded firefly luciferase, were produced as described elsewhere [6], [14], [33]. HIV-1 was used for all pseudotyped viral constructs. Pseudotyped virion-like particle concentrations were determined by ELISA quantitation of HIV-1 p24 core protein according to the manufacturer's instructions (Alliance HIV-1, PerkinElmer, Waltham, MA).

#### Wild type or wild-type-like viruses

Wild-type-like recombinant EBOV (1976 Mayinga variant)-eGFP (GenBank accession number NC002549), and Hendra and Nipah viruses were prepared in BSL4 laboratories, and quantified with a spectrofluorometer or immunoassays, respectively as described elsewhere [6], [34], [35], [36]. Details of the Hendra and Nipah virus variants are reported elsewhere [35], [36]. The WNV replicon plasmid (pWIIrep-GFP) was kindly provided by Dr. Doms (University of Pennsylvania School of Medicine). The coding region of WNV (Strain NY99) capsid (C), premembrane (prM), and envelope (E) proteins was cloned into the pCAGGS.MCS vector. A glycosylation mutant of WNV E protein (N154Q) was introduced using the QuikChange method (Invitrogen). West Nile virion-like particles were produced as previously reported [37], [38] and described in the Methods S1.

### Infection assays

All infections were performed in 96-well white clear bottom cell culture microplates unless indicated otherwise.

#### Pseudotyped virions (single-round infection assays)

Adherent HEK293F and HEK293S GnTI^−^ cells (5×10^3^/100 µl) were incubated with EMEM or DMEM/F12 (2.5% FBS), respectively for 48 hours until 60–70% confluent (approximately 2.5×10^4^/100 µl). THP-1 cells (5×10^4^/100 µl), stimulated with PMA (10 ng/ml) and supplemented with IL-4 (100 ng/ml) to upregulate potential EBOV receptors [39], became differentiated and adherent after 72 hours of incubation in RPMI 1640 (2.5% FBS). Monocyte-derived macrophages were prepared and maintained as described above. The inoculum consisted of 1200 pg p24/100 µl for each viral construct unless indicated otherwise. 1200 pg HIV p24 corresponds approximately to 2×10^7^ HIV-EBOZ GP virion-like particles according to the formula: p24 mass × (1 mol/MW p24 [24,000 g/mol]) × (6.02×10^23^ molecules/1 mol) × (1 virion/2000 molecules). Based on these calculations, the estimated virion-like particle to cell ratio (approximation to MOI) for HEK293F and HEK293S cells was 800∶1, and 400∶1 for THP-1 cells. Before infecting cells, the sera, virion-like particles and/or cells were prepared as follows: 1) MBL-replete or –deficient (LYPB/LYPB; MBL <0.6 ng/ml) sera were preincubated with dilutions of oligosaccharides, mannan, polyethylene glycol, EDTA, 3F8 or isotypic control IgG1 at 37°C for 30 minutes, 2) virion-like particles were preincubated with dilutions of human sera (pretreated as above for certain experiments), rhMBL, cyanovirin-N, PNGase F, endo H, thermolysin or serum-free media at 37°C for 1 hour, or 3) cells were preincubated with dilutions of pharmacologic inhibitors of endocytosis at 37°C for 1 hour, or *N*-glycosylation inhibitors at 37°C for 18 hours, and appropriate controls (dimethyl sulfoxide [DMSO] or methanol for endocytosis inhibitors). Reagents were diluted in DMEM or Veronal-buffered saline (VBS, barbital 2.5 mM, sodium barbital 1.5 mM, NaCl 0.14 M) to achieve optimal rates of infection. The final CaCl_2_ concentration was 5 mM unless indicated otherwise. Media were removed from the cells (except for the media containing pharmacologic inhibitors of endocytosis), and the inoculum preparation was added. Of note, cells preincubated with methyl-β-cyclodextrin were washed twice with phosphate-buffered saline (PBS) before virus was added to obviate disruption of virion glycans or envelopes. Microplates were centrifuged at 1000×*g* for 2 hours at room temperature (spinoculation step), and then incubated at 37°C in 5% CO_2_ for 3 hours. Thereafter, the inoculum preparation was replaced with fresh media containing 10% FBS and incubated at 37°C in 5% CO_2_ for 40 hours.

Cell viability was assessed with 10% alamarBlue reagent (resazurin reduction assay) by measuring fluorescence after 1 hour at 37°C (570 nm excitation, 585 nm emission) in certain experiments when the use of various virion-like particles (e.g. HIV-EBOV, HIV-VSV-G, HIV-*env* negative and HIV-EBOV ΔGP NTDL6) or potentially toxic chemicals (e.g. EDTA, *N*-glycosylation inhibitors) may have differentially impaired cell proliferation. Cells were lysed and single round infection of cells was quantified by detection of chemiluminescence measured with a luciferase assay according to the manufacturer's instructions (Promega, Madison, WI) [6]. Fluorescence and luminescence were read with a Spectramax M5 microplate reader (Molecular Devices, Sunnyvale, CA). Luciferase values were expressed as relative light units per second (RLU/sec) and adjusted for cell viability where appropriate: **Adjusted luciferase value** is defined as luciferase RLU/sec ÷ alamarBlue fluorescence units. Experiments were performed twice in quadruplicate.

#### Wild type-like recombinant EBOV (Mayinga variant)-eGFP virus

We modified a previously described infection assay [6], [34]. Briefly, virions were preincubated with media or 5% MBL-deficient serum (MBL<0.6 ng/ml) with or without 10 µg/ml MBL (final concentration) at 37°C for 1 hour and then the virion preparation was added to 4×10^4^/100 µl HEK293T cells at a multiplicity of infection of 0.1 at 37°C for 1 hour. In certain experiments, MBL was preincubated with mannan or cells were preincubated with tunicamycin as described above. Thereafter, the viral inoculum was replaced with fresh DMEM and incubated at 37°C for 72 hours. Fluorescence was read with a spectrofluorometer (Molecular Devices; excitation, 485 nm; emission, 515 nm). Experiments were performed twice in triplicate.

#### Hendra virus and Nipah virus

The infection assay was described in detail elsewhere [6] with the following modification. Viruses (10,000 TCID_50_/ml) were preincubated with 10% heat-inactivated MBL-deficient serum (MBL<0.6 ng/ml) with or without 10 µg/ml rhMBL at 37°C for 45 minutes before adding the inoculum to Vero E6 cells at 37°C for 1 hour. Experiments were performed twice in duplicate.

#### West Nile virus

1×10^4^ HEK293T cells/well were plated in a 24-well tissue-culture plate in 1 ml DMEM with 10% FBS at 37°C for 24 hours. 250 µl West Nile virion-like particles were preincubated with 50 µl DMEM, 2% MBL-deficient human serum, with or without 10 µg/ml rhMBL at 37°C for 1 hour. Cell culture medium was replaced with 300 µl of West Nile virion-like particle preparation and the plate was spinoculated at 2,000×*g* for 1 hour. The virion-like particle preparation was replaced with 1 ml/well DMEM and 10% FBS, and incubated at 37°C for 24 hours. Cells were detached using TrypLE (Invitrogen) and washed three times with PBS at 4°C. Cells were resuspended in 300 µl PBS and rates of transduction were assayed by flow cytometry. Experiments were performed twice in triplicate.

### Flow cytometry

Various concentrations of FITC-dextran were preincubated with dilutions of rhMBL in RPMI-1640 with 10% FBS at 37°C for 30 minutes. FITC-dextran with or without MBL was then incubated with PMA-stimulated, IL-4 supplemented THP-1 cells (1×10^5^/100 µl reaction volume) in the dark at 37°C for 1 hour. The effect of MBL on FITC-dextran endocytosis and GFP expression in West Nile virion-like particle-transduced HEK293T cells were analyzed with flow cytometry. Data were acquired using a FACSCalibur flow cytometer (BD Biosciences, San Jose, CA) and analyzed using CellQuest Pro software (BD Biosciences). Percentage and mean fluorescent intensity (MFI) of GFP-expressing cells within the live cell population were determined and analyzed as a function of their product (percentage × MFI). Experiments were performed twice in triplicate.

### RNA interference screen

#### pLK0.1 infection

We performed an RNAi loss-of-function screen with high-titer pLKO.1 lentiviral vectors, which carry the puromycin-resistance gene and drive short hairpin RNA (shRNA) expression from a human U6 promoter, to identify potential receptors or attachment factors for EBOV and/or MBL-mediated viral uptake. We obtained 4 to 5 unique shRNA constructs per gene from The RNAi Consortium (Broad Institute, Massachusetts Institute of Technology, MA)[40] targeting 24 candidate lectin, scavenger and other human receptors or receptor-like molecules (Table S1) and cathepsin-L (CTSL1), some of which have been implicated in EBOV pathogenesis (e.g. TYRO3, DC-SIGN, MGL, LSECtin, CTSL1) [41], [42], [43], [44], [45], [46], [47], as well as an empty pLK0.1-puromycin vector. After cultivating HEK293F cells (1.25×10^4^/100 µl) in 96-well microplates in EMEM with 10% FBS at 37°C in 5% CO_2_ for 24 hours (∼60% confluence), adherent cells were transduced in quadruplicate by incubating shRNA viruses and control vectors (4.6×10^8^ particles) with hexadimethrine bromide (6 µg/ml) and cells at 37°C for 18 hours. The medium was replaced with fresh complete medium containing 5 µg/ml puromycin (optimal concentration was determined by a preliminary kill curve experiment) and incubated for 48 hours at which time cell viability was assayed with alamarBlue reagent according to the manufacturer's instructions.

#### HIV-EBOV-GP infection (single round)

We preincubated pseudotyped EBOV virion-like particles (1000 pg p24/100 µl) with or without MBL (10 µg/ml) in VBS at 37°C for 1 hour, and then used the virion-like particles to infect quadruplicate shRNA-infected HEK293F cells maintained in 5 µg/ml puromycin. After 48 hours, we measured luciferase expression as described in *Infection assays* and adjusted the results for cell viability. We calculated percentage change in luciferase activity caused by shRNAs relative to the empty pLK0.1 control vector. A positive hit was defined as ≥66% reduction in infection by at least two shRNA constructs for any particular gene.

### Confirmation of knockdown by western blot

We cultivated HEK293F cells (6.6×10^4^/well) with DMEM and 10% FBS in 6-well format for 24 hours. We then transduced cells with 1.5×10^8^ shRNA viruses identified as hits in the RNAi screen as well as non-targeting shRNA-GFP controls with 6 µg/ml hexadimethrine bromide. After 24 hours at 37°C, we replaced the inoculum preparation with fresh complete media containing 5 µg/ml puromycin and we harvested cells after at least 72 hours. Cellular lysates were extracted from shRNA-treated cells with the Qiagen AllPrep protein kit and protein concentrations were measured using a Nanodrop spectrophotometer. 10 µg lysates were mixed with Laemmli loading buffer and DTT (50 mM final concentration), fractionated on 4–20% gradient minigels (Biorad) and transferred to nitrocellulose membranes. Protein molecular weights were determined using the Invitrogen NOVEX pre-stained protein standard. Membranes were blocked with 2.5% BSA dissolved in PBS-T solution (120 mM NaCl, 2.7 mM KCl, 10 mM phosphate buffer, pH 7.4, 0.1% Tween-20) for 1 hour, followed by incubation with primary antibodies at 4°C overnight. Dilutions of selected antibodies were as following: C1QBP (Santa Cruz) 1∶1000, CLEC6A (Abcam) 1∶1000, CLEC7A (Biovision) 1∶5000, CLEC10A (Novus Biologicals) 1∶500, and actin (Millipore) 1∶5000. Membranes were then triple-washed in PBS-T for 5 minutes, and incubated with appropriate secondary antibodies: goat anti-rabbit IgG-HRP or goat anti-mouse IgG-HRP (both Santa Cruz) at 1∶5000 in PBS-T for 30 minutes. After of triple washing, ECL reagents were applied to the membranes and visualized by autoradiography.

mRNA expression of candidate genes was measured by quantitative real-time PCR as described in detail (see Methods S1 and Table S2).

### Statistics

Normally distributed data were compared with analysis of variance tests, skewed data were analyzed with Mann-Whitney *U* or Kruskal-Wallis tests, and categorical data were compared with Student's t- or Fisher exact tests, where appropriate. The Tukey test was used for all-pairwise multiple comparison procedures. Data in figures are shown as mean ± standard error of the mean. All tests were two-tailed. *p* values <0.05 were considered statistically significant.

## Results

### Complement contributes to differential antiviral effects of human MBL

Mannose-binding lectin is a key host defense molecule that collaborates with complement and other soluble and cell-derived molecules to limit invasion by pathogens [4], [5]. We previously demonstrated that rhMBL effectively inhibited infection of human cells by EBOV GP-pseudotyped lentiviral virions and wild type-like EBOV in the presence of active complement in serum [6], [15]. In the course of those experiments, we were not surprised to confirm that complement was essential for the inhibitory effects of rhMBL. In its absence, we found preliminary evidence suggesting a paradoxical rhMBL-dependent increase in EBOV infection in a variety of human cell lines including 293F cells, which produced highly reproducible results and which potentially bind the EBOV receptor binding region to a greater extent than 293 T cells [48]. Given the wealth of examples where viruses subvert immune defense molecules during virus entry and given the evidence for selective pressure against high-producing MBL haplotypes in human populations, we sought to define the mechanisms by which MBL might enhance infection of human cells by EBOV. We also evaluated the effects of MBL on infection by other glycosylated viruses in low complement states to determine whether this mechanism was relevant for glycosylated viruses in general.

### Recombinant and native human MBL enhance EBOV infection in low complement states

**Figure 1:**
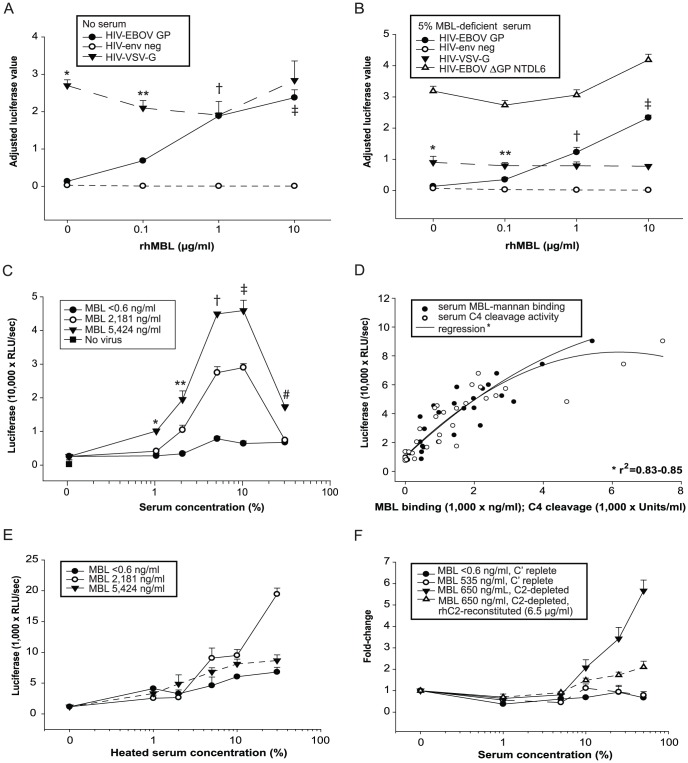
MBL enhances HIV-EBOV GP infection of HEK293F cells in low complement conditions. We used luciferase-encoding HIV-EBOV-GP virion-like particles to infect 5×10^3^ adherent HEK293F cells. Viruses (350 pg p24/100 µl) were preincubated with physiological concentrations of rhMBL (0.1–10 µg/ml) in (A) DMEM or (B) MBL-deficient (<0.6 ng/ml) 5% human serum for 1 hour at 37°C. We analyzed infection rates after 40 hours. Luciferase absorbance values were adjusted for cell viability by normalizing to alamarBlue fluorescence units (resazurin reduction assay) and results are expressed as Adjusted Luciferase Values. Significant differences are shown. * and ** p<0.05 (HIV-VSV-G *vs* both other virions at 0 and 0.1 µg/ml supplemental rhMBL, respectively), † and ‡ p<0.05 (HIV- envelope negative *vs* both other virions at 1 and 10 µg/ml supplemental rhMBL, respectively). Also shown in (B) is the reduced capacity of rhMBL to enhance infection by HIV-EBOV-ΔGP NTDL6 (mutated GP lacks 217 amino acids in the heavily glycosylated mucin-rich region) compared with that for HIV-EBOV GP, which contains intact mucin-rich regions (1.3- vs 17.2−fold enhancement, respectively, p<0.001). Experiments were performed twice in quadruplicate. (C) We preincubated HIV-EBOV GP with serial dilutions of serum from three individuals with undetectable (<0.6 ng/ml; LYPB/LYPB), intermediate (2,181 ng/ml; LYPA/HYPD) or high (5,424 ng/ml; HYPA/LYQA) MBL concentrations. Shown are luciferase values. * p<0.05 (high level MBL *v*s low or intermediate level MBL at 1% serum dilution), **, † and ‡ p<0.05 (all pairwise comparisons at 2%, 5% and 10% serum dilutions, respectively), # p<0.05 (all pairwise comparisons at 30% serum dilution). Experiments were performed twice in quadruplicate. (D) We preincubated HIV-EBOV-GP virion-like particles with 5% serum from 35 ethnically diverse individuals. Shown are associations between level of infection and native MBL activity (mannan-binding or C4 cleavage) for each individual (r^2^ = 0.83, serum C4 cleavage activity; r^2^ = 0.85, serum MBL-mannan binding). Experiments were performed in quadruplicate. (E) We preincubated HIV-EBOV-GP virion-like particles with 5% heat-inactivated serum (56°C for 30 minutes) from three individuals with varying MBL levels before infection of cells. (F) We preincubated HIV-EBOV GP virion-like particles with media or sera (diluted to 1–50%) that were complement component 2 (C2)-replete or depleted, and that lacked or contained approximately equivalent MBL concentrations (535–650 ng/ml). In addition, C2 depleted serum was reconstituted with recombinant human C2 (6.5 µg/ml) for comparison. Experiments were performed twice in quadruplicate.

We first found that HIV-EBOV GP virion-like particles infected HEK293F cells to a greater extent in the presence of MBL than in its absence. RhMBL enhanced infection in a dose-dependent manner either in the absence of serum (Figure 1A) or with a relatively low concentration (5%) of MBL-deficient serum (Figure 1B). In each of these experiments, MBL maximally enhanced viral infection by approximately 18-fold. RhMBL-dependent enhancement of HIV-EBOV GP infection required EBOV glycoprotein since rhMBL did not enhance infection by HIV-*env* negative (gp120^−^) or HIV-VSV-G (Figure 1A, 1B). The permissive effect of rhMBL was calcium-dependent as expected for C-type lectins (Figure S1).

To determine whether native hMBL could also enhance EBOV infections, we collected a panel of human sera with MBL concentrations and C4 cleavage activity that spanned the normal range of MBL variation in human populations (Figure S2). We then tested the relative effects of sera from three individuals with undetectable, intermediate or high MBL levels on the degree of HIV-EBOV GP infection of HEK293F cells. The degree of infection was strongly associated with native MBL levels at serum concentrations ≤10% (Figure 1C). Importantly, EBOV infection was inhibited at serum concentrations >10% in the presence of higher MBL concentrations (Figure 1C) as we reported previously [6]. We validated this association by demonstrating strong correlations between infection and MBL levels using 5% serum from our panel of human sera (MBL concentration curve, r^2^ = 0.85; MBL C4 cleavage activity curve, r^2^ = 0.83; Figure 1D). These findings substantiated our hypothesis that *native* MBL plays a significant role in enhancing EBOV infection.

Given that MBL and activation of the lectin complement pathway are required to block EBOV infection [6], [14], [15], we questioned what effect inactivation of complement would have on MBL's capacity to enhance viral infection. MBL forms a complex with MBL-associated serine proteases (MASPs) and upon binding to pathogens, MBL-MASPs sequentially cleave complement components C4 and C2. The catalytic products of these complement components form the C4b2a complex which acts as the C3 convertase, a key enzyme in the lectin complement pathway. Cleavage and processing of C3 by the C3 convertase produces several C3-derived components (e.g. iC3b) that are potent activators of the immune system. iC3b binds complement receptor 3 (CR3) leading to enhanced phagocytosis by professional phagocytes and increased cytotoxicity by NK cells. Based on the fact that complement is heat labile, we showed that heat inactivation of serum reversed its inhibitory effect on viral infection at higher serum concentrations, which contained higher native MBL levels, albeit to varying extents (Figure 1E). To further define the critical contribution of complement, we compared the effects of human sera from which C2 was removed by immune precipitation and that contained or lacked native MBL (Figure 1F). Notably, C2-depleted MBL-replete serum enhanced infection maximally at a 50% dilution at which MBL's concentration was greatest, an effect that could be reversed by supplementation with recombinant human C2. Complement-replete serum with or without native MBL did not augment infection (Figure 1F).

### MBL-mediated viral infection requires specific glycosylation events known to be essential for its binding interactions

**Figure 2:**
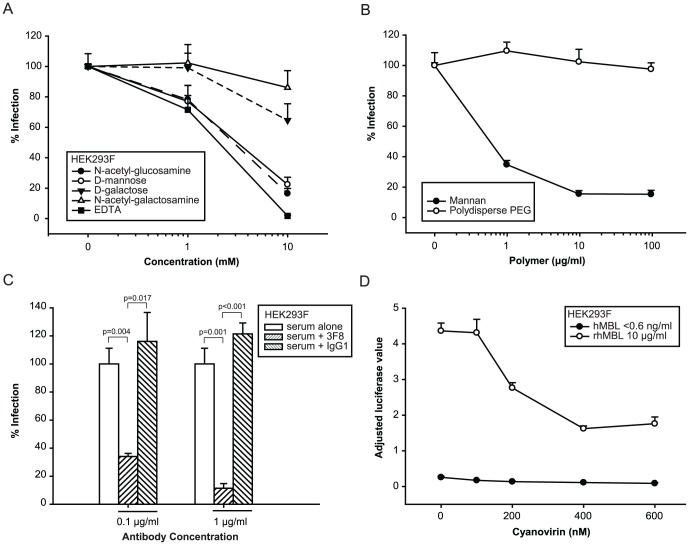
MBL interacts with HIV-EBOV GP via MBL carbohydrate recognition domains. We preincubated 5% serum containing native human MBL (3,621 ng/ml) with (A) 0, 1 and 10 mM of hexose monosaccharides or EDTA diluted in media, or (B) 0–100 µg/ml of mannan or polydisperse polyethylene glycol (PEG)(D) at room temperature for 30 minutes. Then we incubated the serum with HIV-EBOV GP (1200 pg p24/100 µl) at 37°C for 1 hour before infecting adherent HEK293F cells. Luciferase values were adjusted for cell viability using alamarBlue (resazurin reduction assay). We observed relatively more toxicity associated with 10 mM EDTA but this did not invalidate our results because of our adjustment for cell viability. (C) We repeated the previous experiments with 3F8, an anti-human MBL monoclonal antibody or an IgG1 isotype control (preincubation at 37°C for 30 minutes). Significant differences are shown. (D) We preincubated HIV-EBOV GP virion-like particles with cyanovirin (0–600 nM) at 37°C for 1 hour before incubating the particles with 5% serum in the presence or absence of rhMBL. Luciferase values were adjusted for cell viability. Experiments were performed twice in quadruplicate.

Mannose-binding lectin recognizes specific configurations of hexose sugars that include D-mannose, glucose, L-fucose, and *N*-acetylglucosamine but not galactose and sialic acid [6]. To ascertain whether MBL mediates viral infections via the lectin's carbohydrate recognition domain, we preincubated 5% human serum (MBL concentration, 3,621 ng/ml) with competitor carbohydrates. D-mannose, *N*-acetylglucosamine (Figure 2A) and mannan, a polymer of mannose (Figure 2B) reduced HIV-EBOV GP infection, whereas the inhibitory effects of _D_-galactose, *N*-acetylgalactosamine (Figure 2A) and polydisperse polyethylene glycol (Figure 2B) were significantly less or absent. EDTA also reduced infection which is consistent with the MBL carbohydrate recognition domains' dependence on calcium for binding (Figure 2A). In a related experiment, 3F8, an anti-MBL neutralizing monoclonal antibody [31] inhibited HIV-EBOV GP infection (Figure 2C).

To investigate potential viral epitopes targeted by MBL, we used cyanovirin-N (CV-N), a cyanobacterial lectin that binds with high specificity and affinity to Manα1-2Man epitopes at the terminal ends of *N*-linked high-mannose oligosaccharides [49]. CV-N inhibited rhMBL-mediated enhancement of HIV-EBOV GP infection in a dose-dependent manner (IC_50_ = 197 nM) indicating that these lectins either shared a viral epitope or competed via steric hindrance (Figure 2D).

**Figure 3:**
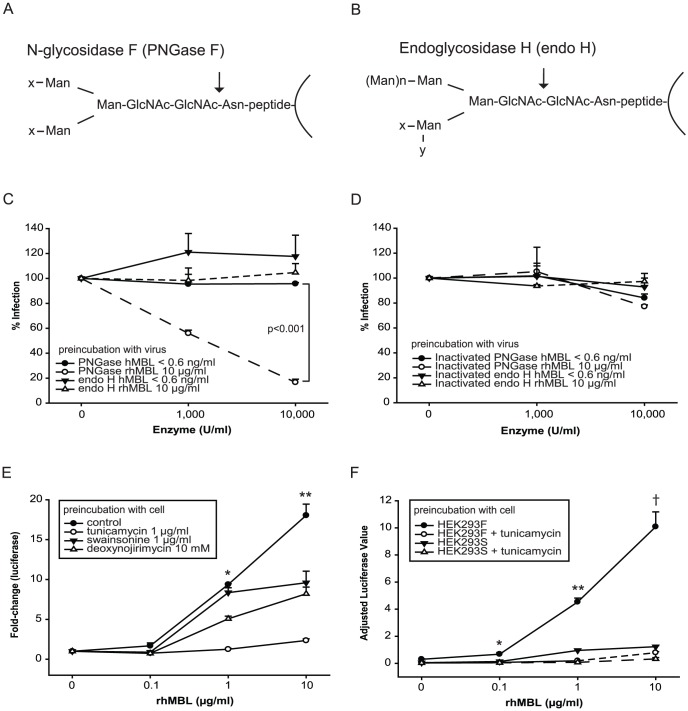
MBL targets *N*-linked glycans on viral and cellular surfaces. The cleavage sites of two endoglycosidases are shown (A,B). N-glycosidase F (PNGase F) is an amidase that cleaves the linkages between the innermost GlcNAc and asparagine residues within high-mannose, hybrid and complex oligosaccharides of *N*-linked glycoproteins, thereby producing carbohydrate-free peptides without any potential ligands for MBL. Endoglycosidase H (endo H) cleaves linkages within the diacetylchitobiose stem of high-mannose of *N*-linked glycoproteins, thereby generating a truncated sugar molecule with one *N*-acetylglucosamine residue (a potential target for MBL) remaining on the asparagine. Man, mannose; GlcNAc, *N*-acetylglucosamine; asn, asparagine; × and y, various oligosaccharides; n = 2–150 residues. We preincubated HIV-EBOV-GP virion-like particles (1200 pg p24/100 µl) at 37°C for 1 hour with (C) PNGase F or endo H (0–10,000 U/ml), or (D) the same concentrations of heat inactivated enzymes. Then we incubated the viruses with 5% MBL-deficient serum in the presence or absence of rhMBL at 37°C for 1 hour before infecting HEK293F cells. Significant differences are shown. (E) We preincubated HEK293F cells at 37°C for 1 hour with chemicals (tunicamycin, swainsonine or deoxynojirimycin) that inhibit various stages of *N*-linked glycosylation. Then we infected cells with HIV-EBOV-GP virion-like particles (1200 pg p24/100 µl) that had been preincubated with 5% MBL-deficient serum and supplemented with various concentrations of rhMBL. Significant differences are shown. * and **, p<0.001 (all pairwise comparisons at 1 and 10 µg/ml rhMBL, respectively). (F) We cultivated HEK293F and HEK293S (deficient in *N*-acetylglucosaminyltransferase I) cells in 5% MBL-deficient serum which was supplemented with various concentrations of rhMBL. We infected cells with HIV-EBOV-GP virion-like particles (1200 pg p24/100 µl) in the absence or presence of 1 µg/ml tunicamycin. Statistical differences among inhibitors at various rhMBL concentrations are shown. *, ** and †, p<0.005 (all pairwise comparisons at 0.1, 1 and 10 µg/ml rhMBL, respectively). Luciferase values were adjusted for cell viability using alamarBlue (resazurin reduction assay). All experiments were performed twice in quadruplicate.

To further explore whether *N*-linked glycans of the viral GP were required for MBL-mediated uptake, we deglycosylated HIV-EBOV GP virion-like particles before adding rhMBL. We compared the effects of two endoglycosidases, PNGase F and endo H which predictably cleave *N*-linked glycans at different sites. Unlike PNGase F, endo H leaves a single GlcNAc residue which is a potential binding site for MBL (Figure 3A, B). First, we confirmed that the endoglycosidases did in fact remove high-mannose moieties from GP of endoglycosidase-treated HIV-EBOV viruses. We proved this with a western blot using biotinylated *Hippeastrum* hybrid (amaryllis) lectin to detect α-mannose residues (the hybrid lectin does not bind to α-glucosyl structures); high-mannose was present in substantial quantities only in untreated, non-deglycosylated virus (Figure S3). We found that PNGase F inhibited infection in a dose-dependent manner in the presence of rhMBL (Figure 3C), whereas heat-inactivated PNGase F did not inhibit infection (Figure 3D). In contrast to this effect, neither active nor heat-inactivated endo H inhibited infection (Figure 3C, D).

We then tested MBL's effect on HIV-EBOV-ΔGP NTDL6 which lacks a portion of the glycosylated mucin-rich region [33] thought to be important for viral pathogenesis [50]. MBL exhibited reduced capacity to enhance infection by HIV-EBOV-ΔGP NTDL6 compared with HIV- EBOV expressing wild type GP (Figure 1B). To confirm these findings, we preincubated HIV-EBOV GP virion-like particles with thermolysin, a bacterial metalloprotease, which cleaves the mucin region and glycan cap of EBOV GPs [46], [48], [51]. In support of our hypothesis that MBL binds the glycan-rich regions, we demonstrated that thermolysin abrogated the MBL-mediated enhancement of HIV-EBOV GP infection in a thermolysin-concentration dependent manner. In the presence of rhMBL, the maximal reduction of viral infection was 17-fold, whereas there was no inhibition of infection in the absence of MBL (Figure S4).

Finally, we investigated whether glycoproteins with *N*-linked glycans on the cell surface were required for MBL-mediated uptake. We demonstrated that MBL-mediated infection was almost completely abrogated by tunicamycin, a chemical inhibitor of *N*-acetylglucosaminyltransferase I (GnTI) which initiates the glycosylation process of cellular glycoproteins (Figure 3E). Preincubation of cells with 1-deoxynojirimicin and swainsonine, which block carbohydrate trimming by inhibiting glucosidase I-II and α-mannosidase II [52] also reduced MBL-mediated infection albeit to a lesser extent than tunicamycin, relative to the control in which MBL enhanced viral uptake by 18-fold (Figure 3E). These chemical inhibitors are potentially cytotoxic but we accounted for differences in cell viability by means of cell proliferation assays (alamarBlue resazurin reduction assays). Similarly, MBL-mediated enhanced infection was not seen in HEK293S cells that are genetically deficient in *N*-acetylglucosaminyltransferase I (Figure 3F).

### MBL mediates EBOV infection via the canonical macropinocytosis-like pathway but distinct endocytic trafficking may define this novel uptake mechanism

**Figure 4:**
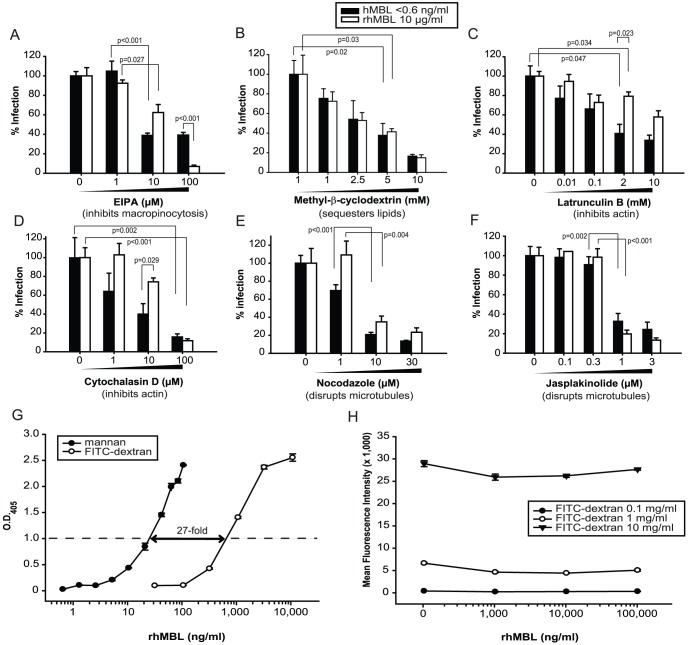
MBL mediates HIV-EBOV GP infection via the canonical macropinocytosis pathway for EBOV but with less dependence on actin. We preincubated HEK293F cells with (A) EIPA (5-(*N*-Ethyl-*N*-isopropyl)amiloride, a potent and specific inhibitor of Na^+^/H^+^ exchanger activity), (B) methyl-β-cyclodextrin (extracts or sequesters cholesterol from the plasma membrane), (C) latrunculin B (blocks actin polymerization), (D) cytochalasin D (inhibits actin microfilament function), (E) nocodazole (disrupts microtubules), or (F) jasplakinolide (disrupts microtubules) in 5% MBL-deficient serum in the absence or presence of rhMBL at 37°C for 1 hour. We then infected cells with HIV-EBOV-GP virion-like particles (1200 pg p24/100 µl). Percentages of infected cells are relative to DMSO controls. Luciferase values were adjusted for cell viability. Experiments were performed twice in quadruplicate. Significant differences are shown. (G) Absorbance values of an ELISA assay are shown indicating the difference in amount of rhMBL within the physiological range that binds to immobilized mannan or FITC-dextran (1 µg/100 µl). (H) We preincubated FITC-dextran with various concentrations of rhMBL at 37°C for 30 minutes and then added the products to PMA-stimulated (10 ng/ml), IL-4-supplemented (100 ng/ml) THP-1 cells at 37°C for 1 hour. We measured FITC-dextran uptake by flow cytometry and reported the results as mean fluorescence intensity (geometric mean fluorescence × percentage of cells). Experiments were performed twice in triplicate.

Canonical EBOV viral entry utilizes a cholesterol-rich lipid raft-dependent macropinocytosis-like pathway [17], [53]. We tested several pharmacological perturbants of endocytosis to investigate whether MBL enhanced viral uptake via this canonical pathway or if it mediated alternative trafficking. The amiloride analog, EIPA which is important for macropinosome formation [54], inhibited infection of HEK293F cells both in the presence or absence of rhMBL indicating that macropinocytosis is the primary mechanism of entry (Figure 4A). Amiloride hydrochloride, which also inhibits macropinocytosis, produced similar results (Figure S5). The absence of a differential effect of methyl-β-cyclodextrin likewise suggested that lipid rafts were required for viral infection regardless of the presence of MBL (Figure 4B). Finally, viral infection was dependent on microtubule function with or without MBL but importantly, MBL reduced the dependence on functional actin (Figure 4C–F).

We confirmed that early endosomal acidification is important for EBOV infection as evidenced by the fact that the lysosomotropic agent bafilomycin A1, a specific and potent inhibitor of vacuolar H^+^ ATPase in early endosomes [55], reduced infection but to a significantly lesser extent in the presence of MBL suggesting that MBL-mediated endocytosis bypasses early endosomes (Figure S5). On the other hand, monensin, a carboxylic ionophore which inhibits both endosomal acidification and receptor recycling, attenuated infection to a similar extent in the presence or absence of MBL (Figure S5).

In agreement with Saeed et al. [17], who used replication-competent infectious EBOV, we found that dynasore, a potent and specific dynamin inhibitor, did not have any significant effect on infection regardless of the presence of MBL (Figure S5). This suggested that vesicle budding which is common to clathrin- and caveolin-mediated endocytosis did not contribute to viral entry in HEK293F cells.

Finally, we tested whether MBL's role in mediating endocytosis was target specific. We found that physiological concentrations of MBL bound effectively to dextran, a prototypic fluid phase marker of macropinocytosis (Figure 4G). However, MBL did not enhance the uptake of dextran (Figure 4H). Therefore, we concluded that MBL enhances EBOV infection in a target-specific manner.

### MBL enhances HIV-EBOV GP infection of cell line-derived and primary human macrophages

**Figure 5:**
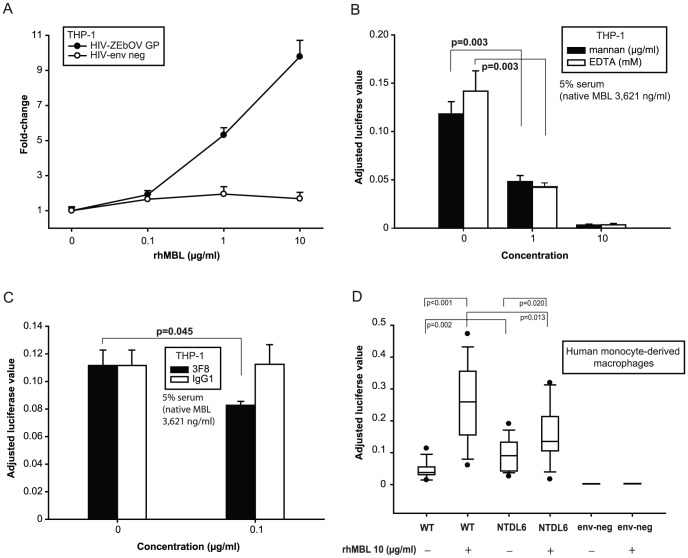
MBL enhances HIV-EBOV GP infection of THP-1 cells and human monocyte-derived macrophages. (A) We stimulated 5×10^4^ THP-1 cells with PMA (10 ng/ml) and supplemented the cells with IL-4 (100 ng/ml) for 72 hours. We preincubated HIV-EBOV GP or HIV-*env* negative virion-like particles (1200 pg p24/100 µl) with or without rhMBL before infecting differentiated adherent THP-1 cells cultivated in 5% MBL-deficient serum. (B) We cultivated 2.5×10^5^ PBMC derived from human single-donor buffy coat samples in RPMI-1640 with 10% FBS and stimulated the cells with M-CSF (50 ng/ml) to induce differentiation of monocyte-derived macrophages. We infected cells with HIV-EBOV GP (WT), HIV-EBOV-ΔGP NTDL6 (NTDL6, mutated GP lacks 217 amino acids in the heavily glycosylated mucin-rich region) or HIV-*env* negative (env neg) in the presence or absence of rhMBL. The box plot represents outliers (dots), 10^th^ and 90^th^ percentiles (whiskers), 25^th^ and 75^th^ percentiles (box) and median values (line). Significant differences in infection rates are shown. Luciferase values were adjusted for cell viability using alamarBlue (resazurin reduction assay) for all the above experiments, which were performed twice in quadruplicate.

To determine whether MBL can enhance viral uptake of EBOV in prototypic target cells (monocytes and macrophages) [12], we tested whether MBL enhanced HIV-EBOV GP infection of activated THP-1 cells (human monocytic cell line) and primary human macrophages. We found that rhMBL enhanced infection of both cell types in low complement conditions (Figure 5A, D). In a separate experiment, infection of THP-1 cells was significantly inhibited by preincubating 5% serum (native MBL concentration, 3,621 ng/ml) with competitors: EDTA (98% reduction with 10 mM; Figure 5B), mannan (97% reduction with 10 µg/ml; Figure 5B) and 3F8 (25% reduction with 0.1 µg/ml; Figure 5C). Furthermore, MBL's capacity to enhance infection was significantly reduced when the EBOV GP mucin-rich region was mutated (Figure 5D).

### EBOV interacts with novel transmembrane lectin receptors

We sought to identify candidate receptors that might be involved in MBL-enhanced EBOV infection. We used short hairpin (sh)RNA-expressing lentiviral constructs to knock down expression of 24 candidate lectin, scavenger and other human cellular receptors (Table S1) in HEK293F cells. We then infected cells with HIV-EBOV GP in quadruplicate in the presence or absence of rhMBL. In the first instance, five different shRNAs targeting the endosomal protease, CTSL1 (Figure S6) reduced infections by 22–99% in the presence or absence of MBL, which lent credence to the validity of our assay because proteolysis of EBOV GP by cathepsin-L in the host cell endosome is a critical step for viral entry [45], [51]. The RNAi Consortium at the Broad Institute previously validated the specificity of all five cathepsin-L shRNAs with gene transcription assays (S. Silver, personal communication). We did not perform a western blot to validate reduced protein expression. We then proceeded to demonstrate that knockdown of MGL (CLEC10A) reduced infections by 47–88% (Figure S6). We verified knockdown of MGL gene expression by measuring mRNA transcripts relative to non-targeting shRNA controls and demonstrated variable reductions of 15%, >95% and 55% for MGL shRNA constructs 2, 3 and 4, respectively (Figure S6; Methods S1). However, in our hands, there were no significant differences in protein expression compared with an empty pLK0.1 control vector (data not shown). We also found a 70% reduction in infection of cells treated with one TYRO3 shRNA which reduced gene expression by 62% but did not reduce protein expression (data not shown). These findings are not conclusive but do not conflict with the roles that these proteins have been reported by others to play in EBOV pathogenesis [43], [47].

**Figure 6:**
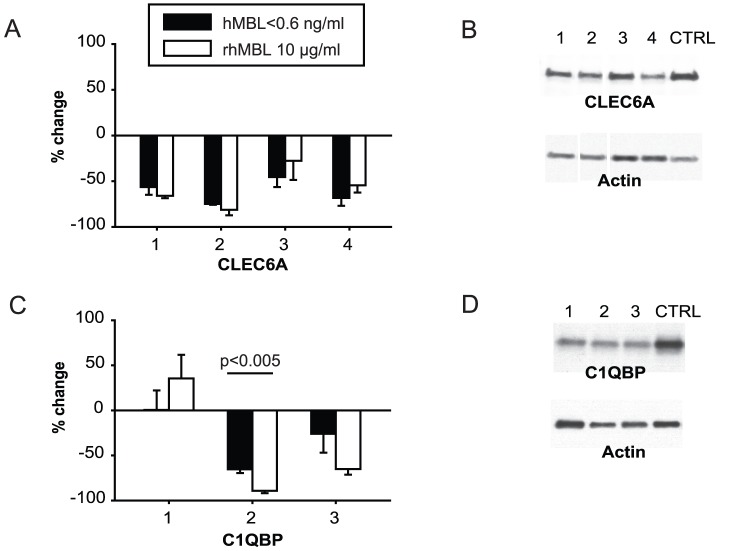
RNA interference screen of candidate cellular receptors for EBOV and MBL. (A–D) We targeted 24 candidate lectin, scavenger and other putative receptors using pLKO.1 lentiviral vectors that expressed 4 or 5 unique short hairpin RNA (shRNA) constructs per gene. We transduced HEK293F cells in quadruplicate using 4.6×10^8^ viral particles (shRNA-expressing vectors or empty control vectors) with hexadimethrine bromide (6 µg/ml) at 37°C for 18 hours. We selected transduced cells with 5 µg/ml puromycin over 48 hours and determined cell viability with alamarBlue reagent (resazurin reduction assay). We then infected cells in quadruplicate with HIV-EBOV GP virion-like particles (1000 pg p24/100 µl) with or without rhMBL. After 48 hours we measured rates of single-round infection (luciferase assay) and adjusted results for cell viability. Percentage change in infection was normalized to the empty pLK0.1 control vector (CTRL). Shown are positive hits (A, C) which were defined as ≥66% reduction in infection by at least two shRNA constructs for any particular gene. Reductions in protein expression produced by shRNAs (western blots; B, D) relative to that produced by the empty pLK0.1 control vector are shown. Relative densitometry was performed with ImageJ (NIH) by adjusting for variations in the actin loading controls (adjusted relative densities for CLEC6A: lane 1, 0.60; lane 2, 0.56; lane 3, 0.51; lane 4, 0.35; control, 1.0. C1QBP: lane 1, 0.32; lane 2, 0.52; lane 3, 0.47; control 1.0).

We then screened the selected candidate attachment factors (Table S1). We demonstrated that shRNA-mediated knockdown of dectin-2 (CLEC6A, dendritic cell-associated C-type lectin 2) reduced HIV-EBOV GP infection regardless of the presence of MBL (Figure 6A) and knockdown of C1QBP (gC1qR) reduced HIV-EBOV GP infection to a significantly greater extent in the presence of rhMBL than in its absence for one of the shRNA constructs (Figure 6C; shRNA construct 2, p<0.005), thereby potentially identifying a novel MBL-mediated viral uptake pathway. Knockdown of protein expression was confirmed by western blots in which single clear bands were detected for CLEC6A (Figure 6B) and for C1QBP (Figure 6D). After adjusting for variations in protein loading relative to actin controls, we confirmed that protein expression of CLEC6A was reduced by 40% to 65% by each of four shRNAs (Figure 6B) and that of C1QBP was reduced by 48% to 68% by each of three shRNAs (Figure 6D) compared with that of the empty pLK0.1 control vector. These experiments validated the findings from the infection assays. We also showed by means of quantitative real-time PCR that CLEC6A gene transcription was knocked down by >95% by each of the same four shRNAs.

### MBL enhances wild type-like EBOV and other glycosylated viral infections

**Figure 7:**
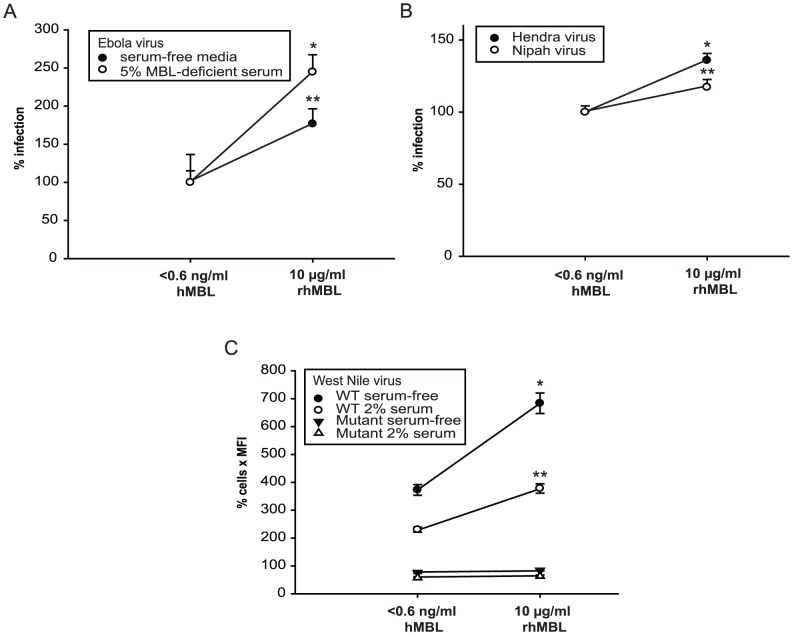
MBL enhances infections by wild type-like EBOV and other glycosylated virions. (A) We preincubated wild type-like EBOV-eGFP (1976 Mayinga variant) with media alone or 5% MBL-deficient serum with or without rhMBL at 37°C for 1 hour and then infected 4×10^4^ HEK293T cells (multiplicity of infection, 0.1) at 37°C for 1 hour. We measured cellular fluorescence after 72 hours of incubation in fresh media. Comparisons are with baseline values, * p = 0.028; ** p = 0.037. (B) We preincubated native Hendra and Nipah viruses (10,000 TCID_50_/ml) with 10% heat-inactivated MBL-deficient serum with or without rhMBL and then infected Vero E6 cells at 37°C for 1 hour. After 24 hours, infection was detected by chemiluminescence-based viral protein immunoassays. Comparisons are with baseline values, *p = 0.001; ** p = 0.029. (C) We preincubated 250 µl West Nile virion-like particle-GFP with media alone or 2% MBL-deficient human serum, with or without rhMBL at 37°C for 1 hour and then transduced 1×10^4^ HEK293T cells. Cells were detached using TrypLE and washed three times with PBS at 4°C. Rates of transduction were assayed by flow cytometry. Comparisons are with baseline values, * p = 0.002; ** p = 0.001. WT refers to wild type; mutant refers to glycosylation mutant of WNV E protein; hMBL refers to human MBL.

Finally, we confirmed that the MBL-enhancement of viral infection was relevant for native viral infections by testing wild type-like EBOV [34]. RhMBL enhanced EBOV infection in low complement conditions by 75 to 150% (Figure 7A). As seen for pseudotyped viruses, MBL-dependent enhanced infection was blocked by preincubating rhMBL with mannan (Figure S7) or pretreatment of cells with tunicamycin in a dose-dependent manner (Figure S7). To evaluate the generalizeability of our observations for other glycosylated viruses, we tested three additional native viral infection models. RhMBL enhanced native Nipah and Hendra viral infections in hypocomplementemic conditions by 20 to 35%, respectively (Figure 7B). In addition, rhMBL significantly enhanced West Nile virion-like particle transduction by 150 to 300% in low complement conditions but it had no effect on a glycosylation-deficient WNV N154Q mutant strain (Figure 7C).

## Discussion

Mannose-binding lectin is a multifunctional pattern recognition receptor that plays a key role in fine-tuning the innate immune system [4]. Since MBL deficiency was first discovered as a cause of a common opsonic defect in children [56], a better understanding of its diverse functions has evolved [5]. MBL binds to invariant glycans on diverse microorganisms including EBOV [6], [14], upregulates opsonophagocytosis, activates the lectin complement pathway, amplifies host responses in cooperation with TLR2/6, and differentially regulates cytokine production [4], [57], [58]. Yet, a cogent explanation for the evolutionary selection of low-producing MBL haplotypes, particularly in indigenous Africans and South Americans, is lacking [9], [10], [59]. We describe for the first time that MBL enhances EBOV, WNV infections, and to a lesser extent Hendra and Nipah viruses in low complement conditions. These findings suggest that certain infectious diseases may have negatively selected high-producing MBL genotypes and they provide new insights in EBOV pathogenesis.

According to the currently accepted paradigm, MBL deficiency predisposes children and immunocompromised individuals to invasive diseases whereas normal or high levels of MBL are protective. In contrast to this, we demonstrated here an apparent paradoxical phenomenon in which recombinant human MBL and native MBL from a panel of human serum samples enhanced pseudotyped and wild type-like EBOV infections of human cells when complement components were reduced or removed (Figure 1). Heat inactivation of serum had a similar albeit diminished effect because heat also reduced C4 cleavage activity of MBL by 39–91% (data not shown).

We demonstrated that MBL binds to EBOV via highly specific interactions with MBL's carbohydrate recognition domains, a process which is calcium-dependent as would be expected for C-type lectins (Figure S1). We further showed that high affinity MBL ligands (e.g. D-mannose [6]) competed with viral-binding to MBL (Figure 2A,B), and 3F8, a neutralizing monoclonal antibody that binds the hinge region within the MBL carbohydrate recognition domain [31] blocked enhanced infection (Figure 2C).

We then sought to identify which specific residues on the viral surface were targeted by MBL. Based on the repertoire of MBL's typical ligands, we hypothesized that the targets were glycoproteins, which are critical for virus stability, cell tropism, immune evasion, and host cell invasion. EBOV, and other viruses such as Hendra and Nipah viruses, WNV, hepatitis C virus, HIV-1, HIV-2, influenza viruses, metapneumoviruses, and SARS-corona virus use host cell machinery to modify viral surface proteins by *N*-linked glycosylation. This process involves attaching a high-mannose core to the amide nitrogen of asparagines followed by trimming and remodeling of viral oligosaccharides in the endoplasmic reticulum and Golgi apparatus [60]. EBOV GPs are heavily glycosylated with *N-* and *O*-linked glycans that constitute more than one-third of their molecular mass [61]. The majority of *N*-linked glycans are concentrated in the GP1 glycan cap and mucin-like region, which are reminiscent of the glycan shields of HIV gp120 and Epstein-Barr virus gp350. Other investigators have speculated that glycans may help EBOV evade immune responses by masking the putative EBOV receptor-binding region [62], [63]. But viral glycans may also be recognized by C-type lectin receptors that are either membrane-associated (e.g. DC-SIGN [42]) or soluble (e.g. MBL [6]), and several viruses can hijack these type of molecules to enter cells and propagate [60].

We established that MBL binds to *N*-linked glycans on the viral surface by showing that cyanovirin-N (a lectin that targets *N*-linked high-mannose with great specificity and affinity) competed with MBL binding to viruses (Figure 2D) [49]. Furthermore, MBL-enhanced HIV-EBOV GP infection was significantly diminished after deglycosylation of virions using PNGase F [50], [64] but not endo H, which leaves an exposed *N*-acetylglucosamine residue on the polypeptide chain that may serve as a ligand for MBL (Figure 3C,D). These findings were confirmed by the fact that MBL-mediated enhancement of viral infection was abrogated by thermolysin-treated HIV-EBOV GP virions in a thermolysin-concentration dependent manner (Figure S4). MBL also had a significantly reduced capacity to enhance EBOV infection when the GP lacked a portion of the glycosylated mucin-rich region (Figure 1B). It is noteworthy, however, that infection levels of EBOV with mutated GP were higher than those of wild-type GP pseudotypes, likely because absence of the GP's mucin-rich region exposed the putative EBOV receptor binding region which facilitated infection of cells [33]. On the other hand, MBL did not enhance infection by VSV-G pseudotyped virions indicating to some extent MBL's selectivity.

These findings raised the question whether MBL can cross-link the virus to putative cell surface receptors. It is known that EBOV protects itself from host immunity by down-regulating host proteins including immune regulatory molecules and receptors such as MHC1 and β1 integrins via steric occlusion, referred to as the “glycan umbrella” effect [50], [65]. To circumvent this constraint on infection, we propose that EBOV may exploit putative MBL receptors to enter cells with or without engaging EBOV-specific receptors. Although the cognate receptor/s for MBL are not known with certainty, potential receptors for MBL include CD93 (C1qRp), CD35 (complement receptor 1, CR1), calreticulin (cC1qR), CD91, and the binding protein for the globular domain of C1q (gC1qBP/gC1qR/C1QBP) [4], [28], all of which have proven and/or predicted *N*-linked glycopeptides [66], [67]. Therefore, we tested whether reduction of *N*-linked glycoprotein receptor expression [68] by chemical (tunicamycin) or genetic (HEK293S GnTI^−^ cells) approaches could reduce MBL-mediated HIV-EBOV GP infection. Tunicamycin, which completely inhibits *N*-linked glycosylation, abrogated MBL-mediated pseudotyped EBOV infection to a greater extent than other downstream *N*-glycan-trimming agents such as deoxynojirimycin and swainsonine which leave residual mannose or GlcNAc residues (Figure 3E). Similarly, HEK293S *N*-linked glycosylation-deficient cells were resistant to MBL-mediated enhancement (Figure 3F). Taken together, these results showed that MBL interacts with *N*-linked glycoproteins trafficked via the ER and Golgi apparatus. This finding is corroborated by the fact that the collagenous stalk of MBL binds to calreticulin, a putative receptor [27] and lung collectins (surfactant proteins A and D) can also bind ligands via their collagenous tails [69].

The mechanisms of EBOV attachment and internalization have been studied extensively [17], [19], [45], [47], [50], [53], [70], [71], [72], [73], [74], [75]. EBOV attaches to various putative attachment factors on the cell surface [13] where they undergo either clathrin/caveolae/dynamin-independent, actin/lipid raft-dependent macropinocytosis-like endocytosis [17], [70], [74] or clathrin/caveolae/dynamin-dependent endocytosis [75] depending on type of target cells involved. Virions are then trafficked through early and late endosomes where they undergo proteolysis in acidified endocytic vesicles by cathepsin L and/or B which remove the mucin-like region of GP leading to enhanced viral binding and infectivity [17], [19], [33], [45], [46], [47], [70]. Previous studies used replication-competent infectious EBOV, EBOV virion-like particles or pseudotyped lentiviruses. Findings from our infection assay using EBOV GP pseudotyped lentiviruses agreed with those of Saeed et al., who used wild type viruses, to the extent that EBOV undergoes macropinocytosis-like endocytosis (Figure 4A) in a lipid raft-dependent (Figure 4B) and dynamin-independent (Figure S5) manner [17], [74]. In addition, we found that MBL diminished the dependence on actin filaments (but not microtubules) and early endosomal acidification [70], [76] for infection (Figure 4C-F, Figure S5), suggesting that MBL may traffic viruses via a non-canonical pathway that resembles the clathrin/caveolin/dynamin/actin-independent, and microtubule-dependent endocytic pathway utilized by lymphocytic choriomeningitis virus (LCMV) and recombinant LCMV-Lassa virus GP infectious virions [77]. Further detailed mechanistic studies are required to confirm these findings.

To determine if the phenomenon of MBL-dependent enhancement is applicable to clinically relevant targets, we tested a human monocyte-derived macrophage cell line (Figure 5A) and primary human macrophages (Figure 5B) which represent the type of cells targeted early in the course of natural EBOV infection. MBL did indeed enhance HIV-EBOV GP infection in both types of cells.

We then sought to identify cellular surface receptor(s) that mediated MBL-enhanced viral infection. We conducted an RNAi screen using at least 4 unique shRNA-expressing vectors per gene to mitigate the impact of differential effectiveness and possibility of off-target effects of shRNAs [40]. We tested our assay by comparing levels of HIV-EBOV GP infection after knocking down CTSL1 (Figure S6)[47] and the reduced infectivity was consistent with the known role of CTSL1 in mediating viral entry. Using the same techniques, we revealed that knock down of C1QBP by three shRNA constructs (confirmed by western blot, Figure 6D) reduced HIV-EBOV GP infection to variable extents in two of the three shRNA treatments (variability of shRNAs is well described). Notably, knock down of C1QBP by both shRNAs was augmented in the presence of MBL but to a significantly greater extent for the most effective shRNA (65% to 90% reduction of infection, p<0.005) suggesting that MBL plays a supplementary role in mediating viral entry (Figure 6C, D). C1QBP is a tetrameric transmembrane surface protein expressed on human monocytes and a variety of other cells. It binds the globular heads of C1q via its amino terminus with high affinity and contains three potential *N-*glycosylation sites [78]. C1QBP has been implicated in innate and adaptive immunity. Viral ligands have been shown to activate C1QBP translocation which inhibits RIG-1, an RNA helicase, which in turn inhibits antiviral signaling by downregulating type I interferons [79]. Given that induction of RIG-1 inhibits EBOV replication [80] we speculate that EBOV-MBL complexes activate C1QBP which then negatively regulates RIG-1 inhibition of viral infection, thereby enhancing viral proliferation. However, it is not clear how C1QBP avoids binding to circulating MBL under normal physiological conditions. One hypothesis is that MBL undergoes a conformational change upon binding to viral and other targets [81]. This in turn could expose potential binding sites in MBL's collagenous stalk for engagement with C1QBP.

Furthermore, we showed that knock down of the C-type lectin receptor, dectin-2 reduces experimental infection with EBOV (Figure 6A). Dectin-2 is expressed on certain macrophages, monocytes and dendritic cells [82], which are targeted by EBOV early in the course of infection. This receptor has not been invoked previously in viral pathogenesis. Further studies will be required to determine if there are specific physical and/or functional interactions between EBOV, MBL and dectin-2, and to determine if this receptor plays a role in native EBOV infections.

Finally, we investigated whether MBL enhances native EBOV and certain other glycosylated virus infections in low complement conditions. RhMBL did indeed enhance infection by wild type-like EBOV (by 75–150%), West Nile virion-like particles (by 150–300%), and native Nipah and Hendra viruses (by only 20–35%, respectively) suggesting that other viruses that are adorned with *N-*glycans can also be potentiated by MBL to varying extents (Figure 7A–C). As expected, MBL-enhancement of EBOV infection was mitigated by mannan and tunicamycin (Figure S7) and infection with a WNV glycosylation mutant was not enhanced by MBL (Figure 7C). It is plausible that life-threatening glycosylated viruses that form glycosylated virions from the Old World such as measles and variola viruses exerted pressure on the evolution of MBL genotypes that was not previously appreciated.

In support of our hypothesis that MBL enhances infections caused by certain glycosylated virions, there is evidence that individuals with high MBL concentrations or high-producing MBL haplotypes experience enhanced infections caused by other glycosylated intracellular pathogens such as *Leishmania* spp, *Mycobacterium leprosum* and *M. tuberculosis*, and opportunistic organisms in HIV-infected individuals [10], [83], [84], [85], [86]. Tobin et al. [87] identified mutations in zebrafish and human leukotriene A4 hydrolase genes that conferred heterozygous advantage against *Mycobacterium marinum* and *tuberculosis*, respectively. These results set a groundbreaking precedent in our understanding of how SNPs in a single human gene can dictate dichotomous inflammatory states: either inadequate or excessive immune responses can result in increased severity of TB meningitis in humans. Similarly, we speculate that under certain circumstances both high and low levels of MBL may predispose individuals to certain infectious diseases. Furthermore, our hypothesis that high levels of MBL may be deleterious is supported conceptually by a recent report that demonstrated a role for MBL in mediating immune pathology in a mouse model of Ross River virus (RRV) arthritis; high MBL levels in serum and synovial fluid of humans with RRV correlated with disease severity [88]. MBL has also been implicated in murine models of pandemic H1N1 influenza A and H9N2 avian influenza A as a risk factor for severe disease by upregulating inflammatory responses [89]. There is also evidence that inhibition of the lectin pathway confers considerable protection against non-infectious disorders such myocardial and gastrointestinal ischemia/reperfusion injury [90]. Therefore, it is possible that evolutionary selection of mutant MBL alleles conferred protection against a variety of pathogens and/or reduced the deleterious effects of complement-mediated inflammation [8], [11], [83], [84], [85]. Other examples of preserved heterozygozity include sickle-cell trait which protects against malaria despite its disadvantage in healthy populations. Also, a persistent regulatory mutation in the Duffy antigen chemokine receptor confers resistance to malaria.

Wide variations in plasma concentrations of MBL and certain complement components occur naturally. These variations are due to diversity of genotypes of MBL and complement component C4, which is encoded by 2 to 8 copies of the C4 gene [91], as well as other complement components. Partial C4 deficiency is one of the most common immune protein deficiencies in humans. It is caused by a complex pattern of differences in gene size, gene number, and nucleotide polymorphisms. Specifically, homoexpression or deletion of C4A or C4B is estimated to occur in 19–34% of normal Caucasians [92], [93]. Total C4A deficiency occurs in 1% and C4B deficiency in 10% of the Finnish population [94]. The mean C4 protein levels vary between 0.26 and 0.49 g/L in European Americans and Asian Indian populations [93], [95]. However, it is notable that total C4 protein levels span a 1-log range (approximately 0.1 to 1.0 g/L) with higher concentrations correlating with a larger number of gene copies [93], [95]. Although the prevalence of homozygous C2 deficiency in Caucasians is rare (approximately 1/20,000), partial C2 deficiency is estimated to affect about 1% of western Europeans [96]. In addition, certain infectious diseases may modulate complement levels e.g. acute malaria is a pervasive ancient infectious disease that can cause hypocomplementemia [97], [98]. Furthermore, complement may be reduced to varying extents by certain auto-immune conditions that drive immune complex formation, although these events are relatively rare. To investigate our hypothesis that relatively low levels of complement may permit MBL to enhance viral infection, we performed infection assays in serially diluted serum. We observed that serum containing higher concentrations of native MBL enhanced HIV-EBOV GP infection to the greatest extent when serum was diluted by at least 10-fold (Figure 1C). We then showed that the critical factor in serum (diluted only 2-fold) that abrogated MBL's capacity to enhance infection is complement (specifically C2; Figure 1F). It is in this context that we modeled viral infections using high level rhMBL (10 µg/ml) in 5% serum (i.e. diluted 20-fold). However, such a high concentration of rhMBL is considered supraphysiological if adjusted for the 20-fold dilution factor of serum.

Although human MBL is produced primarily in the liver, extra-hepatic MBL synthesis has been found in the gastrointestinal tract, middle ear fluid, and nasopharyngeal secretions. It is possible that *MBL2* expression can be differentially induced in various compartments in response to different stimuli [99]. Similarly, extrahepatic complement synthesis and regulation may influence local concentrations of complement [100]. Furthermore, dysfunctional MASP-2 variants may attenuate its capacity to activate complement [101]. The frequency of deficient or non-functional MASP-2 ranges from 2% to 19% in different populations with the highest values in Africans [102]. We speculate that *relative MBL excess in the setting of relatively low C4 levels or deficient MASP-2* may lead to MBL-mediated enhanced viral infections in clinical settings. Thus, we propose that MBL, C4 and MASP-2, among others contribute to a lattice of immune molecules that represent an individual's “innate immune haplotype”. Individually, these effectors may not necessarily dictate the outcome of infections, but the aggregate immunological state may influence a person's susceptibility or resistance to infection.

**Figure 8:**
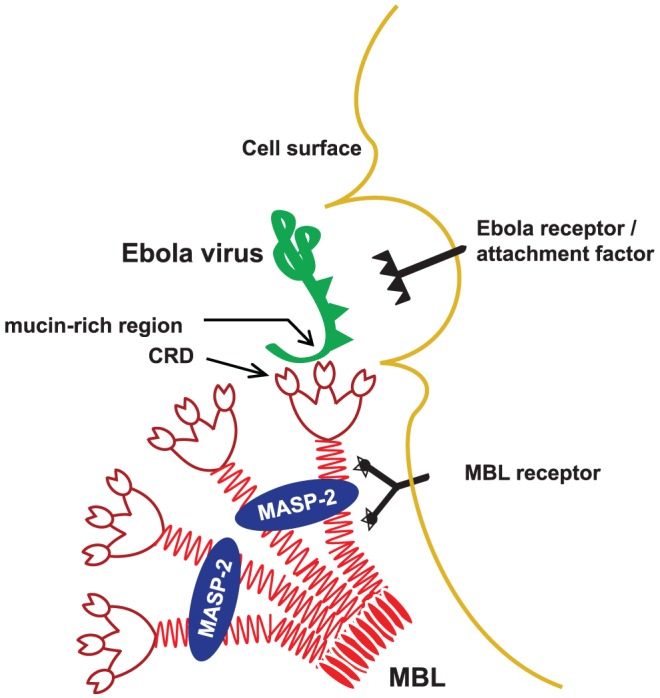
Proposed model of MBL-mediated macropinocytosis of EBOV. MBL carbohydrate recognition domains (CRD) bind to highly glycosylated mucin-rich regions of EBOV GP and the MBL-virion complex is presented to the cell surface. Then MBL binds to cognate cellular receptors, such as C1QBP or calreticulin [6] via MBL collagenous stalks. In this manner, MBL concentrates virus at the cell surface and may facilitate cross-linking of EBOV to its cognate receptor or to other attachment factors such as dectin-2, which may facilitate subsequent membrane fusion and internalization. The relatively large size of EBOV particles (800 to 1,400 nm) is amenable to bulk fluid-phase uptake pathways such as macropinocytosis which is the canonical pathway for EBOV entry. Our data indicate that MBL also mediates internalization of virus via macropinocytosis but suggests that MBL-mediated uptake preferentially utilizes microtubules compared with the canonical EBOV pathway which is dependent on both microtubules and actin.

The precise mechanism by which MBL enhances infections by glycosylated virions is not known. We speculate that MBL concentrates virus at the target cell surface under certain circumstances, such as when MBL exceeds complement levels, and facilitates cross-linking of EBOV to its cognate receptor or to other attachment factors such as MGL or dectin-2, which may facilitate subsequent membrane fusion and internalization or macropinocytosis (Figure 8).

Our study was potentially limited by the fact that we used pseudotyped viruses for mechanistic experiments because the composition of viral envelopes depends in part on the cell type in which viruses are produced. Therefore, the observed interactions between HIV-EBOV-GP and host attachment factors may be artificial. However, we showed a similar phenomenon with wild type-like EBOV and other glycosylated viruses. Furthermore, it has been shown that all the GPs of all the Ebolaviruses (EBOV and relatives) contain high-mannose and hybrid *N*-linked glycans independent of the cell line used for viral propagation [64]. Another limitation of our study was the lack of direct evidence from humans that complement components (specifically C4 or C2) are sufficiently low *in vivo* in certain circumstances to permit MBL to mediate enhanced uptake of glycosylated viruses. We used media supplemented with 5% human serum which is only half the concentration of fetal calf or fetal bovine serum typically used for EBOV infectivity assays [17], [18], [44], [45], [67]. Given that C4 gene copy number variations may lead to a proportionate reduction of circulating C4 [88], we speculate that the experimental ratios of MBL:C4 used in this study are physiologically plausible. A recent clinical study by Kainulainen et al. [94] provided preliminary data which are consistent with our hypothesis that synergy between C4 deficiency and MBL excess may predispose children and adolescents to certain infections: 26% (n = 22) of Finnish patients with recurrent respiratory infections vs.14% (n = 10) of healthy controls (p = 0.048) had C4A deficiency. Not unexpectedly, 50% of the patients with C4A deficiency had MBL concentrations >1.5 µg/ml, the mean human MBL serum concentration but notably, more than one-third had very high MBL levels (>4 µg/ml). Further clinical studies are required to investigate the significance of this apparent association.

In conclusion, this study reveals MBL's complex role in infections caused by EBOV and other glycosylated viruses. MBL, which is a key innate immune molecule, seemingly has paradoxical functions depending on the levels of complement [6], [15]. We demonstrate for the first time the phenomenon of MBL-dependent enhancement of infections caused by glycosylated virions in low complement conditions and discover roles for C1QBP and dectin-2 in EBOV pathogenesis. We postulate that an individual's innate immune haplotype is defined by the interactions between the genotypes of MBL, C4 and possibly others. Our findings highlight the possibility that administration of supraphysiological doses of MBL products or blood products with high MBL concentrations to individuals in the setting of infectious diseases and relative hypocomplementemia may be deleterious under certain conditions. On the other hand, it is intriguing to consider that MBL might be used as an adjuvant to enhance delivery of glycosylated vaccine epitopes to effectors of the immune system. Finally, these findings suggest that the pressure of infectious diseases may have driven, in part, the evolutionary selection of MBL mutant haplotypes that encode low or intermediate MBL serum levels in the majority of humans.

## Supporting Information

Figure S1 **RhMBL enhances HIV-EBOV GP infection in a calcium-dependent manner.** We preincubated HIV-EBOV-GP virion-like particles with rhMBL in 5% MBL-deficient serum and Veronal-buffered saline with or without calcium supplementation. *, ** and † p<0.05 (5 mM CaCl_2_ versus 0 or 2 mM CaCl_2_ at 0, 0.1 and 1 µg/ml rhMBL, respectively); ‡ p<0.001 (all pairwise comparison at 10 µg/ml rhMBL). Experiments were performed twice in quadruplicate.
(EPS)

Figure S2 **MBL activity in sera from a panel of healthy subjects.** We measured native MBL concentrations (mannan-binding activity) and MBL C4 cleavage activity in sera from 35 healthy adult volunteers of Caucasian, Asian, Hispanic or African-American ancestry (females, n = 20). The sample had a 3.7 log_10_ range of MBL concentrations (0–5,424 ng/mL) that were strongly correlated with the concordant MBL C4 cleavage values (0–7,468 U/mL; r^2^ = 0.91). The sample included three individuals with homozygous *MBL2* null alleles (B, C, D) who had no detectable MBL (lower limits of detection: MBL concentration, 0.6 ng/ml; C4 cleavage activity, 1.6 U/ml). As expected, significantly more individuals with wild-type *MBL2* haplotypes (A/A) had MBL concentration >500 ng/ml (12/21 vs 2/14, p<0.03) and MBL C4 cleavage activity >500 U/mL (13/21 vs 1/14, p<0.005) compared with *MBL2* O/O or O/A haplotypes (O refers to B,C or D alleles).
(EPS)

Figure S3 **Endoglycosidases cleave** ***N*** **-linked glycans in HIV-EBOV GP.** We preincubated HIV-EBOZ GP virion-like particles (12,000 pg/ml) with PNGase F or endo H (10,000 U/ml each) diluted in DMEM or with DMEM alone for 1 hour at 37°C. Viruses then underwent gel electrophoresis; products were transferred onto a nitrocellulose membrane and were detected by further incubation with biotinylated *Hippeastrum* Hybrid Lectin (1:500) followed by streptavidin-horseradish peroxidase (HRP; 1:5000). Membranes were then incubated with Immobilon Western Chemiluminescent AP substrate and exposed to autoradiography film. High-mannose moieties were preserved only in untreated virus confirming that PNGase F and endo H effectively cleaved high-mannose residues on GPs of HIV-EBOV virion-like particles.
(EPS)

Figure S4 **Thermolysin treatment of HIV-EBOV GP abrogates enhancement of infection by rhMBL in a thermolysin-concentration dependent manner.** We preincubated HIV-EBOV GP virion-like particles (1200 pg p24/100 µL) without or with thermolysin (31.3, 62.5, 125, 250 µg/ml) in buffer (40 mM HEPES, 40 mM MES, 50 mM NaCl, 0.1 mM CaCl_2_) shaking for 30 minutes at 37°C. The reactions were then terminated with 0.1 mM EDTA. HEK293F cells were infected with thermolysin-treated virion-like particles mixed with MBL-deficient (<0.6 ng/ml) 5% human serum alone or with the same supplemented with 10 µg/ml rhMBL as described in Materials and Methods. Luciferase values were adjusted for cell viability using alamarBlue (resazurin reduction assay). Shown are average adjusted luciferase results from two experiments each with four replicates. The maximal reduction of viral infection was 17-fold in the presence of rhMBL, whereas there was no inhibition of infection in the absence of MBL.
(EPS)

Figure S5 **MBL-mediated macropinocytosis of HIV-EBOV GP may bypass early endosomes and is dynamin independent.** We preincubated HEK293F cells with (A) amiloride hydrochloride, an inhibitor of Na^+^/H^+^ exchanger activity, (B) bafilomycin A1, a specific and potent inhibitor of vacuolar H^+^ ATPase in early endosomes, (C) monensin, an inhibitor of both endosomal acidification and receptor recycling, and (D) dynasore, a potent and specific dynamin inhibitor (which is involved in clathrin- and caveolin-mediated endocytosis) in 5% MBL-deficient serum in the absence or presence of rhMBL at 37°C for 1 hour. We then infected cells with HIV-EBOV-GP virion-like particles (1200 pg p24/100 µl). Percentages of infected cells are relative to DMSO controls (a methanol control was used for monensin). Luciferase values were adjusted for cell viability using alamarBlue (resazurin reduction assay). Significant differences are shown. Experiments were performed twice in quadruplicate.
(EPS)

Figure S6 **shRNA knockdown of cathepsin-L (CTSL1) and MGL (CLEC10A).** We performed RNA interference with a pLK0.1 vector encoding shRNAs that targeted (A) CTSL1 and (B) MGL (CLEC10A) as described for Figure 6. Luciferase values were adjusted for cell viability. Percentage change in infection was normalized to an empty pLK0.1 control vector.
(EPS)

Figure S7 **rhMBL targets** ***N*** **-linked glycans on surfaces of wild type-like EBOV and HEK293T cells.** (A) We preincubated 5% MBL-deficient serum (with or without MBL supplementation) with media or mannan (1 µg/ml) at 37°C for 1 hour. Then we incubated wild type-like EBOV-eGFP (1976 Mayinga variant) with the above preparations at 37°C for 1 hour. Finally, we infected 4×10^4^ HEK293T cells with virions at a multiplicity of infection of 0.1. (B) We preincubated (i) HEK293T cells with varying concentrations of tunicamycin and (ii) wild type-like EBOV-eGFP with 5% MBL-deficient serum (with or without rhMBL supplementation) at 37°C for 1 hour. Then we infected 4×10^4^ HEK293T cells with virus at a multiplicity of infection of 0.1. Fluorescence was assayed 72 hours after infection. Significant differences are shown. Experiments were performed in triplicate.
(EPS)

Table S1 **List of human genes included in RNA interference (RNAi) screen.**
(DOC)

Table S2 **Primers for qRT-PCR assays.**
(DOC)

Methods S1 **Supplementary methods.**
(DOC)
