# Supplementary material for: Lectin-Dependent Enhancement of Ebola Virus Infection via Soluble and Transmembrane C-type Lectin Receptors
Source: PLoS One. 2013 Apr 2;8(4):e60838. doi: 10.1371/journal.pone.0060838 (PMC3614905; doi:10.1371/journal.pone.0060838)
Supplement: Table S1 — List of human genes included in RNA interference (RNAi) screen. (DOC) [file pone.0060838.s008.doc]

|  |  |
| --- | --- |
|  |  |
| **Table S1.** List of human genes included in RNA interference (RNAi) screen. | |
|  |  |
| **Gene Symbol** | **Other designations** |
|  |  |
| *Lectin receptors* |  |
|  |  |
| ASGR2 | Asialoglycoprotein receptor 2; hepatic lectin H2; C-type lectin domain family 4, member H2 (CLEC4H2) |
| CD93 | Complement component 1 q subcomponent receptor 1 (C1qR1); C1q/mannose-binding lectin/surfactant protein-A receptor |
| CD207 | Langerin; C-type lectin domain family 4, member K (CLEC4K); Langerhans cell specific C-type lectin |
| CD209 | Dendritic cell-specific intracellular adhesion molecules (ICAM)-3 grabbing non-integrin (DC-SIGN); |
|  | C-type lectin domain family 4, member L (CLEC4L); HIV gpl20-binding protein |
| CLEC4G | C-type lectin superfamily 4, member G; liver and lymph node sinusoidal endothelial cell C-type lectin (LSECtin) |
| CLEC4M | C-type lectin domain family 4, member M; CD209 antigen-like protein 1; dendritic cell-specific ICAM-3-grabbing non-integrin-related protein (DC-SIGNR); liver/lymph node-specific ICAM-3 grabbing non-integrin (L-SIGN) |
| CLEC6A | C-type lectin domain family 6, member A; dendritic cell-associated C-type lectin 2; dectin-2 |
| CLEC7A | C-type lectin domain family 7, member A; dendritic cell-associated C-type lectin 1; dectin-1 |
| CLEC10A | C-type lectin domain family 10, member A; macrophage lectin 2 (calcium dependent); CD301; macrophage galactose/*N*-acetylgalactosamine (GalNAc) specific lectin (MGL); HML |
| LGALS1 | Lectin, galactoside-binding, soluble, 1; galectin-1; lactose-binding lectin 1 |
| LY75 | Lymphocyte antigen 75; C-type lectin domain family 13, member B (CLEC13B); DEC-205; CD205; |
| MRC1 | Mannose receptor, C-type 1; macrophage mannose receptor 1; C-type lectin domain family 13, member D (CLEC13D); CD206 |
|  |  |
| *Scavenger receptors* |  |
|  |  |
| CD36 | Scavenger receptor class B, member 3 (SCARB3); collagen type I receptor, thrombospondin receptor; glycoprotein IIIb |
| MSR1 | Macrophage scavenger receptor 1; macrophage acetylated LDL receptor I and II; scavenger receptor class A, member 1 (SCARA1); scavenger receptor class A (SR-A); CD204 |
| OLR1 | Oxidised low density lipoprotein (lectin-like) receptor 1; scavenger receptor class E, member 1; LOX1; |
|  | C-type lectin domain family 8, member A (CLEC8A) |
| SCARF1 | Scavenger receptor class F, member 1; acetyl LDL receptor; scavenger receptor expressed by endothelial cells |
| STAB1 | Stabilin 1; common lymphatic endothelial and vascular endothelial receptor-1 (CLEVER-1); fasciclin, EGF-like, laminin-type EGF-like and link domain-containing scavenger receptor 1 (FELE-1); MS-1 antigen |
|  |  |
| *Other receptors or receptor-like molecules* |  |
|  |  |
| C1QBP | Complement component 1 q subcomponent-binding protein; C1q globular domain-binding protein; gC1qR; glycoprotein gC1qBP; hyaluronan-binding protein 1 |
|  |  |
| CALR | Calreticulin; CRT; cC1qR (receptor for the collagen domain of C1q) ; calregulin |
| CD14 | Myeloid cell-specific leucine-rich glycoprotein |
| CR1 | Complement component (3b/4b) receptor 1; complement receptor type 1; CD35 |
| LRP1 | Low density lipoprotein receptor-related protein 1; alpha-2-macroglobulin receptor; CD91 |
| LRP2 | Low density lipoprotein receptor-related protein 2; megalin; Heymann nephritis antigen homolog |
| TYRO3 | TYRO3 protein tyrosine kinase; tyrosine-protein kinase DTK; tyrosine-protein kinase RSE; tyrosine-protein kinase SKY; tyrosine-protein kinase byk |
