# Supplementary material for: Lectin-Dependent Enhancement of Ebola Virus Infection via Soluble and Transmembrane C-type Lectin Receptors
Source: PLoS One. 2013 Apr 2;8(4):e60838. doi: 10.1371/journal.pone.0060838 (PMC3614905; doi:10.1371/journal.pone.0060838)
Supplement: Table S2 — Primers for qRT-PCR assays. (DOC) [file pone.0060838.s009.doc]

**Table S2. Primers for qRT-PCR assays.**

| **Gene Symbol** | **Oligonucleotide sequence (5’ to 3’)** | **Primer orientation** |
| --- | --- | --- |
| C1QBP | ATCAACTCCCAATTTCGTGGTT | sense |
|  | GGTGGTCATATAAGGCCCAGT | antisense |
| CLEC6A | CAAGAGCAGCAACCTCAAAG | sense |
|  | TTCACTGAAGCAGGTGAGAC | antisense |
| CLEC7A | GAAGGGAGAGGCTGTGATTTC | sense |
|  | TGTCCACAGACAGTCATCTC | antisense |
| CLEC10A | AGCAACTTCACCTCAAACACTG | sense |
|  | CCTCCACCTCAGCTTTCAGA | antisense |
| TYRO3 | CAGGTCTGAAGCTCATGGGAG | sense |
|  | TGGCACACCTTCTACCGTGA | antisense |
| Actin | GCTCG TCGTCGACAACGGCTC | sense |
|  | CAAACATGATCTGGGTCATCTTCTC | antisense |
