# Supplementary material for: Lectin-Dependent Enhancement of Ebola Virus Infection via Soluble and Transmembrane C-type Lectin Receptors
Source: PLoS One. 2013 Apr 2;8(4):e60838. doi: 10.1371/journal.pone.0060838 (PMC3614905; doi:10.1371/journal.pone.0060838)
Supplement: Methods S1 — Supplementary methods. (DOC) [file pone.0060838.s010.doc]

**Supplementary Methods**

***MBL2* genotyping.**

Primers were obtained from the MGH DNA Core Facility. The H/HY forward primer that detects a polymorphism in the promoter region was modified as follows: 5'-GCTTACCCAGGCAAGGGCCTGTG-3'. The reactions were carried out in a final volume of 20 µl containing 50 ng (exon 1), 100 ng (promoter) or 125 ng (5’ UTR) genomic DNA templates, 0.2 mM dNTPs, CaCl_2_ (exon 1, 1.1mM; promoter, 1.7 mM; 5’-UTR, 1.4 mM) and 20 pmol of each primer (controls: DRB1, 20 pmol; hHGH, 25 pmol) run in an Eppendorf Mastercycler pro using Platinum Taq DNA Polymerase and appropriate buffer (Invitrogen). We modified the published thermocycler programs [1] as follows (25 cycles each): exon 1, 20 seconds (s) at 94**°**C, 20 s at 59**°**C, 30 s at 72**°**C; promoter, 30 s at 94**°**C, 30 s at 59**°**C, 45 s at 72**°**C; and 5’-UTR, 30 s at 94**°**C, 30 s at 68**°**C, and 45 s at 72**°**C.

***Hippeastrum* Hybrid Lectin western blot.**

HIV-EBOV GP virion-like particles (12,000 pg/ml) were incubated with 10,000 U/ml Endo H or PNGase F diluted in DMEM or with DMEM alone for 1 hour shaking at 37°C. Treated virus was then boiled for 5 minutes and loaded onto a 4-12% Bis-Tris Gel and run at 150v for one hour. Gels were transferred onto a nitrocellulose membrane (0.45µm) at 100v for 1 hour. Membranes were then blocked (SuperBlock Blocking Buffer, Thermo Scientific) at room temperature for 30 minutes. Membranes were washed in TBS-T three times. The membrane was incubated with biotinylated *Hippeastrum* Hybrid Lectin (1:500; Vector Laboratories, Burlingame, CA) diluted in PBS for 30 minutes. After washing, membranes were incubated with streptavidin-horseradish peroxidase (1:5000) diluted in TBS-T for one hour and then washed again. Membranes were incubated with Immobilon Western Chemiluminescent AP substrate (Millipore) according to manufacturer’s specifications and exposed to autoradiography film.

**Preparation of West Nile virion-like particles.**

Briefly, HEK293T cells plated at 50% confluence in T75 culture flasks were transfected using the calcium phosphate method with 10 µg pCAGGS.MCS-based plasmid DNA encoding wild type or N154Q mutant WNV C, preM, and E proteins and 20 µg of the pWIIrep-GFP plasmid. Six hours later, HEK293T cells were washed once with PBS and maintained in DMEM with 10% FBS. The culture supernatant containing West Nile virion-like particles was harvested 48 hours after transfection. All viruses were stored in aliquots at −80**°**C.

**Quantitative real-time PCR (qRT-PCR).**

RNA was extracted from cell lysates using the Allprep RNA kit (Qiagen) and DNAse I (Invitrogen) according to the manufacturers’ instructions. mRNA expression of candidate genes was measured by quantitative real-time PCR. We synthesized cDNA using SuperScript III First-Strand Synthesis Supermix (Invitrogen). We performed triplicate qRT-PCR for each gene with the iQ SYBR Green Supermix kit (Bio-Rad Laboratories, Hercules, CA) using an Eppendorf Mastercycler ep *realplex*^2^. Primers for genes of interest and β actin were purchased from Invitrogen (Supplementary Table 2). The thermocycler conditions were as follows: 50°C for 2 minutes, 90°C for 10 minutes, then [95°C for 15 s, 60°C for 30 s, 72°C for 30 s] x 50 cycles, followed by 60°C for 15 s, 20 minute melting curve, and 95°C for 15 s. We analyzed expression of the genes of interest relative to β actin (the internal control) in shRNA-targeted samples compared with non-targeting shRNA-GFP control samples using the comparative *C*_T_ method. Specifically, we calculated fold-change in gene expression and percentage knockdown using the formula 2^−ΔΔ^*^C^*^T^ [2]. Efficiencies of the primers for β actin and the genes of interest were similar based on similar logarithmic PCR amplification plots.

**Reference**

1. Steffensen R, Thiel S, Varming K, Jersild C, Jensenius JC (2000) Detection of structural gene mutations and promoter polymorphisms in the mannan-binding lectin (MBL) gene by polymerase chain reaction with sequence-specific primers. J Immunol Methods 241: 33-42.

2. Schmittgen TD, Livak KJ (2008) Analyzing real-time PCR data by the comparative C(T) method. Nat Protoc 3: 1101-1108.
